# Objective assessment of symptomatic vitreous floaters using optical coherence tomography: a case report

**DOI:** 10.1186/s12886-015-0003-5

**Published:** 2015-03-08

**Authors:** Kevin Patrick Kennelly, James Plunkett Morgan, David Jude Keegan, Paul Patrick Connell

**Affiliations:** Mater Misericordiae University Hospital, Dublin, Ireland

**Keywords:** B-scan ultrasound, Optical coherence tomography, Pars plana vitrectomy, Vitreous cyst, Vitreous floaters

## Abstract

**Background:**

Vitrectomy for symptomatic vitreous floaters carries significant risks. Justification of surgery is difficult, particularly in healthy eyes with normal visual acuity and without a posterior vitreous detachment. This is the first reported case of optical coherence tomography being utilized to objectively assess the impact of a vitreous opacity on the macula.

**Case presentation:**

A 37-year-old Caucasian female complained of the sudden onset of a ring-like floater in the central visual field of her left eye. Visual acuity was 20/20, there was no intraocular inflammation and the posterior vitreous was not detached. Complete blood count with differential, serology screen (including cysticercosis and echinococcus), chest x-ray and abdominal ultrasound found no evidence of systemic infective or cystic disease.

A color photograph and B-scan ultrasound confirmed a 4.31 mm free-floating semi-translucent vitreous cyst with a hyperechogenic, pigmented surface and faint internal strands suspended in the mid-vitreous cavity, in the visual axis. The cyst moved with ocular movements, but only within the vitreous lacuna it resided in. Humphrey and Goldmann visual fields were normal. However, spectral domain optical coherence tomography (OCT) demonstrated shadowing on either side of the fovea, consistent with the ring-like scotoma described by the patient. Removing the retinal layers from the 3D-reconstructed macular cube OCT revealed a circular shadow on the macula.

The patient elected for conservative management and at 3-month follow-up her symptoms had almost fully resolved as the cyst migrated to the inferior vitreous cavity, no longer casting a shadow on the macula.

**Conclusion:**

To our knowledge, this is the first description of using OCT as an objective, qualitative assessment of symptoms caused by large vitreous opacities and may provide a simple yet useful adjunctive tool in evaluating the risk-benefit ratio of vitrectomy in patients with large symptomatic vitreous floaters.

## Background

Patients with symptomatic vitreous floaters are often highly motivated for surgery [1]. Neodymium YAG laser vitreolysis has been used to treat vitreous floaters [2,3], but is less efficacious than vitrectomy which completely resolves symptoms in 93% of eyes [4]. However, vitrectomy carries vision-threatening risks including retinal breaks and detachment, proliferative vitreoretinopathy, choroidal haemorrhage, macular pucker, and cataract progression [5]. Justifying floaterectomy without an objective assessment of patients’ symptoms can be difficult, particularly in healthy eyes with normal visual acuity and without a posterior vitreous detachment. Optical coherence tomography (OCT) is a noninvasive imaging technique that has revolutionized the practice of ophthalmology since its introduction in 1991 [6]. OCT has evolved to enable detailed imaging of many intraocular structures [7], however this case report describes the first use of OCT as a means of objectively and qualitatively assessing the visual impact of vitreous opacities. We utilized a large vitreous cyst to illustrate the technique.

## Case presentation

A 37-year-old Caucasian female presented to the ophthalmology department complaining of a six-day history of a ring-like floater in the central visual field of her left eye. She was otherwise systemically well. She had no relevant past medical or ophthalmic history. Visual acuity was 20/20 in each eye. Both anterior segments and the posterior segment of the right eye were normal. Slit lamp biomicroscopy with a 90-diopter lens revealed a large semi-translucent cyst with a pigmented surface free-floating in the mid-vitreous cavity of the left eye. The fundus was normal, there was no intraocular inflammation and the posterior vitreous was not detached.

Complete blood count with differential, serology screen (including cysticercosis and echinococcus), chest x-ray and abdominal ultrasound found no evidence of systemic infective or cystic disease.

A diagnosis of a primary peripheral cyst of the pigmented iris epithelium was made, based on Shield’s classification [8]. The cyst had presumably become symptomatic after being dislodged and migrating to the vitreous cavity.

**Figure 1:**
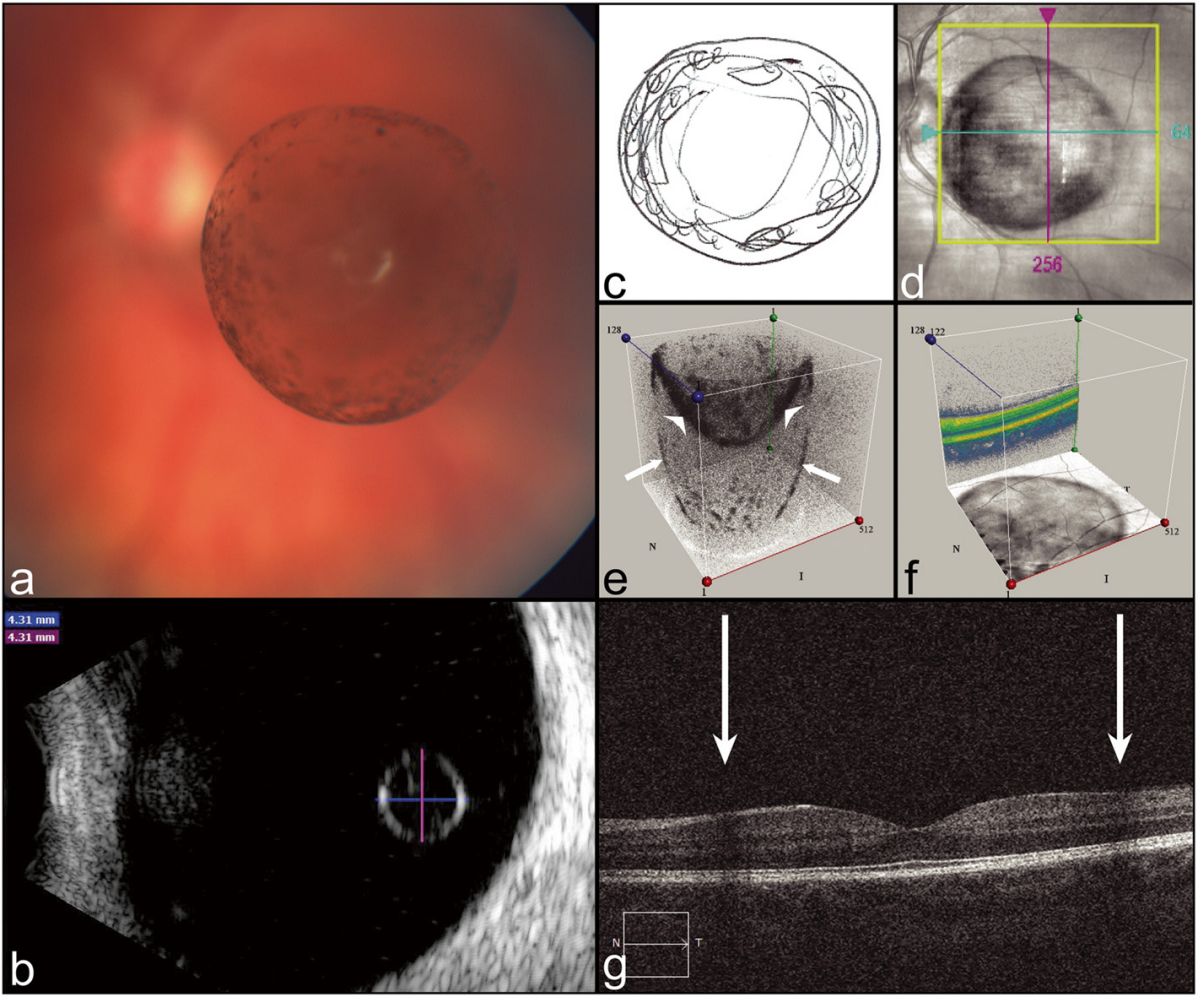
**Objective assessment of a free-floating pigmented vitreous cyst. (a)** Color fundus photograph of the left eye demonstrating a free-floating semi-translucent vitreous cyst with a pigmented surface. **(b)** B-scan ultrasound depicts a hyperechogenic 4.31 mm cyst. **(c)** Patient’s sketch of her ring-like floater. **(d)** En face macular OCT obscured by the cyst. **(e)** OCT through the cyst reconstructed in 3D demonstrates an oval shadow (arrows) cast behind the spherical cyst (arrowheads). **(f)** Macular cube OCT reconstructed in 3D with the retinal layers removed reveals a spherical shadow cast on the macula. **(g)** Cross-sectional OCT demonstrating a shadow on either side of the fovea.

A high-quality color photograph (FF450*plus* Fundus Camera with VISUPAC**®** image processing system, Carl Zeiss Meditec, Germany) (Figure 1a) and B-scan ultrasound (Scanmate DGH8000, DGH Technology Inc., USA) (Figure 1b) confirmed a 4.31 mm vitreous cyst with a hyperechogenic, pigmented surface and faint internal strands suspended in the mid-vitreous cavity, in the visual axis. The cyst moved with ocular movements, but only within the vitreous lacuna it resided in. Humphrey and Goldmann visual fields were normal. However, spectral domain OCT (Cirrus™ HD-OCT, Carl Zeiss Meditec, Germany) (Figure 1d-g) demonstrated shadowing on either side of the fovea (Figure 1g), consistent with the ring-like scotoma described by the patient (Figure 1c). Removing the retinal layers from the 3D-reconstructed macular cube OCT revealed a circular shadow cast on the macula (Figure 1f).

The benign nature of the cyst [8] was explained to the patient and the risks and benefits of vitrectomy [5] discussed. The images presented were used to aid the discussion, and the possibility of the cyst moving from the central visual axis was explained. The patient elected for conservative management and at 3-month follow-up her symptoms had almost fully resolved as the cyst migrated to the inferior vitreous cavity, thereby no longer casting a shadow on the macula.

## Conclusions

While many cases of vitreous cysts have been described, the color photograph presented here provides an outstanding illustration. Using multimodal imaging, we were able to reassure the patient that her visual symptom was secondary to the cyst. Two publications describe the structure of vitreous cysts using OCT [9,10]. OCT has recently been utilized to quantify vitreous signal intensity in patients with uveitis [11]. However to our knowledge, this is the first description of using OCT to illustrate the shadowing effect on the retina by a vitreous cyst, or indeed any large vitreous opacity. This technique enables an objective, qualitative assessment of the symptoms caused by large vitreous opacities and may provide a simple yet useful adjunctive tool in evaluating the risk-benefit ratio of vitrectomy in patients with large symptomatic vitreous floaters.

## Consent

Written informed consent was obtained from the patient for publication of this Case report and any accompanying images. A copy of the written consent is available for review by the Editor of this journal.

## Abbreviation

OCT: Optical coherence tomography
